# Concomitant febuxostat enhances methotrexate-induced hepatotoxicity by inhibiting breast cancer resistance protein

**DOI:** 10.1038/s41598-019-56900-2

**Published:** 2019-12-30

**Authors:** Kenji Ikemura, Shun-ichi Hiramatsu, Yuri Shinogi, Yusuke Nakatani, Isao Tawara, Takuya Iwamoto, Naoyuki Katayama, Masahiro Okuda

**Affiliations:** 1 Department of Pharmacy, Mie University Hospital, Tsu, Mie 514-8507 Japan; 2 Department of Clinical Pharmacy and Biopharmaceutics, Mie University Graduate School of Medicine, Tsu, Mie 514-8507 Japan; 3 Department of Hematology and Oncology, Mie University Graduate School of Medicine, Tsu, Mie 514-8507 Japan; 4 Department of Pharmacy, Osaka University Hospital, Suita, Osaka, 565-0871 Japan

**Keywords:** Chemotherapy, Translational research, Cancer, Adverse effects

## Abstract

Methotrexate (MTX) is an antifolate agent used for the treatment of various malignancies and is eliminated by breast cancer resistance protein (BCRP). Because febuxostat (FBX) is known to inhibit BCRP activity, FBX might exacerbate MTX-related adverse effects. In this study, we examined the drug-drug interaction between FBX and MTX in BCRP-expressing membrane vesicles. Moreover, we retrospectively investigated the impact of FBX on MTX-related adverse effects in 38 patients (144 cycles) receiving high-dose MTX therapy (HDMTX). The Food and Drug Administration Adverse Event Reporting System (FAERS) database and human hepatocellular carcinoma cell line HepG2 cells were used to evaluate the effects of FBX on MTX-induced hepatotoxicity. In the membrane vesicle study, FBX significantly inhibited BCRP-mediated transport of MTX. Concomitant FBX significantly increased the incidence of hepatotoxicity, but not of nephrotoxicity and hematological toxicity in patients receiving HDMTX. FAERS database analyses revealed that the reporting odds ratio of FBX for MTX-induced hepatotoxicity was 4.16 (95% CI: 2.89–5.98). Co-incubated FBX significantly decreased the cell viability and increased cytotoxicity in MTX-treated HepG2 cells. These findings suggest that concomitant FBX enhances MTX-induced hepatotoxicity by inhibiting hepatic BCRP. These findings provide important information for the safe management of HDMTX therapy in clinical settings.

## Introduction

Methotrexate (MTX) is an antifolate agent that inhibits dihydrofolate reductase. High-dose MTX (HDMTX), defined as a dose of ≥1 g/m^2^, is widely used for the treatment of various malignancies, including osteosarcoma, acute lymphoblastic leukemia, and lymphoma^1^. The major adverse effects of MTX include nephrotoxicity, hepatotoxicity, and myelosuppression^2^. Since the serious adverse effects of MTX often correlate with drug exposure level, therapeutic drug monitoring of MTX is essential to prevent its severe toxicities in patients receiving HDMTX^3–5^.

MTX is mainly eliminated from the kidney. Human organic anion transporters hOAT1 (*SLC22A6*) and hOAT3 (*SLC22A8*) are predominantly responsible for the basolateral uptake of MTX in the proximal tubular cells^6,7^. Moreover, ATP-binding cassette transporters, such as breast cancer resistance protein (BCRP/*ABCG2*) and multidrug resistance-associated protein 2 (MRP2/*ABCC2*) are also involved in the excretion of MTX into the urine through the brush-border membrane of proximal tubules^8–11^. In the liver, organic anion transporting polypeptide 1B1 (OATP1B1/*SLCO1B1*) and 1B3 (OATP1B3/*SLCO1B3*) are highly expressed in the hepatic sinusoidal membrane, where they have key roles in the hepatic uptake of MTX^12,13^. Besides, it is known that BCRP and MRP2 expressed in the hepatic canalicular membrane are responsible for the biliary excretion of MTX^8,10,14,15^. It has been reported that co-administration of proton pump inhibitors (PPIs) and non-steroidal anti-inflammatory drugs (NSAIDs), which are well-investigated as inhibitors of OATs, MRP2, and BCRP, delay the elimination half-life of MTX in patients receiving HDMTX^16–19^, which may lead to the increased risk of severe adverse effects.

Febuxostat (FBX), a non-purine selective xanthine oxidase inhibitor, is effective for the prevention of hyperuricemia accompanied by tumor lysis syndrome (TLS) during cancer chemotherapy^20^. Since TLS occurs frequently in patients with hematological malignancies, most of the patients who received HDMTX were also co-administered FBX^21^. Recently, Miyata *et al*.^22^ demonstrated that FBX potently inhibits human BCRP activity. Considering this finding, we hypothesized that concomitant FBX may affect the severity of MTX-related adverse effects by inhibiting the transport of MTX via BCRP. In fact, the hepatotoxicity of MTX is well known to be caused by hepatic accumulation of MTX^23^. However, the drug-drug interaction between FBX and MTX and the impact of FBX on the development of MTX-related adverse effects remains to be explored in clinical settings.

In the present study, we investigated drug-drug interaction between FBX and MTX with BCRP-expressing plasma membrane vesicles. Besides, the impact of concomitant FBX on the development of MTX-related adverse effects was examined by retrospective chart review of the hospitalized patients who received HDMTX therapy, database analysis using Food and Drug Administration (FDA) Adverse Event Reporting System (FAERS), and an *in vitro* study using human hepatocellular carcinoma cell line (HepG2 cells).

## Results

### Inhibition of BCRP-mediated uptake of [^3^H]MTX by FBX

**Figure 1:**
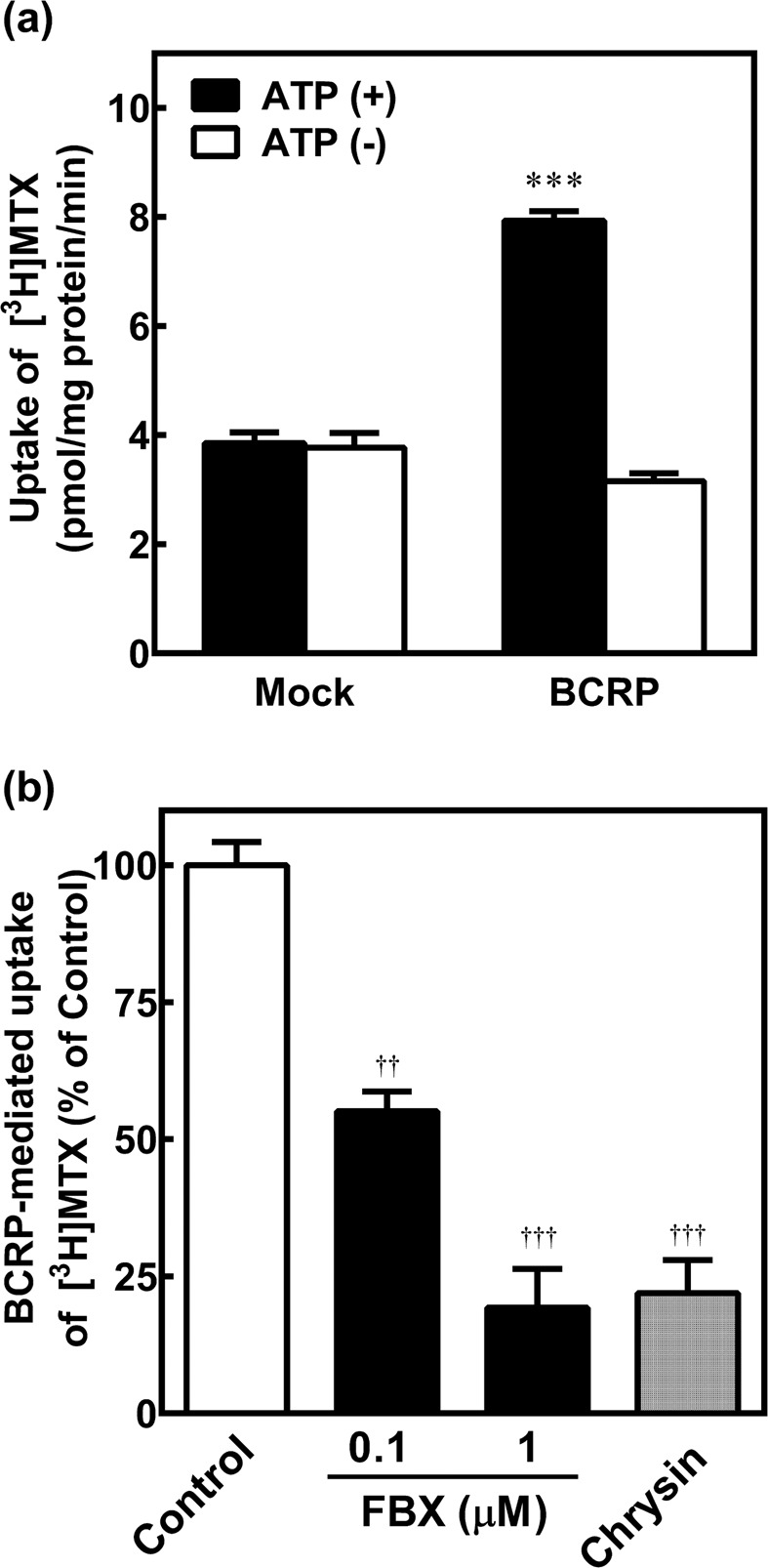
Effect of FBX on BCRP-mediated uptake of [^3^H]MTX. (**a**) The uptake of [^3^H]MTX (10 µM) was estimated for 5 min with human BCRP-expressing plasma membrane vesicles and mock-transfected vesicles in the presence or absence of ATP. (**b**) The BCRP-mediated uptake of [^3^H]MTX (10 µM) using BCRP-expressing membrane vesicles was estimated for 5 min in the presence or absence of 1 μM chrysin and 0.1 or 1 μM FBX. BCRP-mediated [^3^H]MTX uptake was calculated by subtracting the [^3^H]MTX uptake in the mock-transfected with ATP from that in the BCRP-expressing vesicles with ATP. Each column represents the mean ± S.E. of three separate experiments. Statistical analyses were performed using the Dunnett’s test. ^***^p < 0.001 compared to mock-transfected vesicles with ATP, ^††^*p* < 0.01, ^†††^*p* < 0.001 compared to control (vehicle).

To verify whether [^3^H]MTX is specifically transported by BCRP, the uptake of [^3^H]MTX (10 µM) was measured for 5 min with the human BCRP-expressing plasma membrane vesicles and the mock-transfected vesicles in the presence or absence of ATP (Fig. 1a). We confirmed the linearity in the uptake of [^3^H]MTX up to 10 min in the BCRP-expressing vesicles in the presence of ATP. As shown in Fig. 1a, the uptake of [^3^H]MTX in the BCRP-expressing vesicles was significantly higher than that in the mock-transfected vesicles in the presence of ATP (*p* < 0.001), and this increased uptake was not observed in an ATP free condition.

To assess whether FBX inhibits BCRP-mediated transport of [^3^H]MTX, the uptake of [^3^H]MTX (10 µM) with BCRP-expressing membrane vesicles was measured for 5 min in the presence or absence of 1 μM chrysin (a potent BCRP inhibitor) and 0.1 or 1 μM FBX (Fig. 1b). Chrysin potently inhibited the BCRP-mediated uptake of [^3^H]MTX. In addition, the BCRP-mediated uptake of [^3^H]MTX was decreased to 55% and 19% of that of the control by co-incubation of 0.1 and 1 μM FBX, respectively.

### Characteristics of the included patients

After considering the inclusion and exclusion criteria, 38 patients (HDMTX administration: 144 cycles) were enrolled in this study. The characteristics of the included patients are summarized in Table 1. Twenty-four patients (72 cycles) received FBX during therapy with HDMTX. The potential drug interactions with MTX were verified using Lexi-Interact^TM^ for all the study patients. Drug interaction between MTX and PPI was identified in 14 (19%) and 34 (47%) cycles in patients receiving and not receiving FBX, respectively. The rate of co-administered PPI was significantly higher in patients not receiving FBX than those receiving FBX (*p* < 0.001). The other characteristics were not significantly different between patients receiving and not receiving FBX.

**Table 1:** Characteristics of the included patients.

	Without FBX (72 cycles, n = 16)	With FBX (72 cycles, n = 24)	*p*-value
Female	39 (54)	30 (42)	0.182
Age (years)	63 [45–78]	63 [36–74]	0.789
Body weight (kg)	57.5 [26.5–83.6]	55.8 [34.3–80.4]	0.598
Body surface area (m^2^)	1.46 [1.03–2.03]	1.57 [0.85–2.05]	0.855
MTX dose (g/m^2^)	3.5 [3.0–3.5]	3.5 [2.8–3.5]	0.325
Baseline biological parameters			
AST (U/L)	19 [10–33]	19 [11–39]	0.787
ALT (U/L)	19 [6–97]	15 [6–88]	0.818
ALP (U/L)	276 [120–532]	242 [140–1578]	0.169
D-Bil (mg/dL)	0.1 [0.1–0.2]	0.1 [0.1–0.2]	0.405
Scr (mg/dL)	0.59 [0.39–0.99]	0.64 [0.38–1.02]	0.796
LDH (IU/L)	220 [77–627]	195 [151–525]	0.283
ANC (×10^9^/L)	3.41 [0.52–6.44]	3.62 [0.69–8.30]	0.154
Hb (g/dL)	10.0 [7.2–14.6]	10.2 [7.1–14.4]	0.985
PLT (×10^9^/L)	209 [24–615]	283 [89–615]	0.170
Co-administered drugs			
Proton pump inhibitors	34 (47)	14 (19)	<0.001
Hepatoprotective agents	6 (8)	9 (13)	0.587
G-CSF agents	27 (38)	18 (25)	0.150

Values are presented as the median [range] or cycles (%). n: number of patients.

Fisher’s exact test or Mann–Whitney U test was performed.

ALP: alkaline phosphatase, ALT: alanine transaminase, ANC: absolute neutrophil count, AST: aspartate transaminase, D-Bil: direct-bilirubin, FBX: febuxostat, G-CSF: granulocyte-colony stimulating factor, Hb: hemoglobin, LDH: lactate dehydrogenase, MTX: methotrexate, PLT: platelet.

### Hepatotoxicity, nephrotoxicity, and hematological toxicity following HDMTX therapy in patients receiving and not receiving FBX

The number of patients showing hepatotoxicity, nephrotoxicity, and hematological toxicity in the two groups is summarized in Table 2. The incidence of hepatotoxicity in patients receiving FBX (17%) was significantly higher than in those not receiving FBX (3%, *p* = 0.009). Moreover, the characteristics of hepatotoxicity in cases defined as acute drug-induced liver injury are summarized in Supplementary Table 1. In all cases, the clinical patterns of hepatotoxicity were hepatocellular injury. The causalities of MTX for hepatotoxicity were classified as “highly probable” or “probable” whereas those of FBX were “unlikely” or “excluded”. On the other hand, there were no significant differences in the incidence of nephrotoxicity and hematological toxicity between the two groups (*p* = 1.000).

**Table 2:** Hepatotoxicity, nephrotoxicity, and hematological toxicity in patients with and without receiving FBX after HDMTX therapy.

	Without FBX (72 cycles, n = 16)	With FBX (72 cycles, n = 24)	*p*-value
Hepatotoxicity	2 (3), n = 1	12 (17), n = 9	0.009
Nephrotoxicity	1 (1), n = 1	1 (1), n = 1	1.000
Hematological toxicity	32 (44), n = 14	31 (43), n = 17	1.000
Anemia	1 (1)	1 (1)	
Neutropenia	31 (43)	25 (35)	
Thrombocytopenia	14 (19)	6 (8)	

Values are presented as cycles (%). n: number of patients.

Fisher’s exact test was performed.

Nephrotoxicity and hematological toxicity were evaluated according to CTCAE ver. 5.0.

FBX: Febuxostat, HDMTX: high-dose methotrexate.

### Comparison of serum MTX concentrations at 24 h, 48 h, and 72 h following HDMTX therapy in patients receiving and not receiving FBX

**Figure 2:**
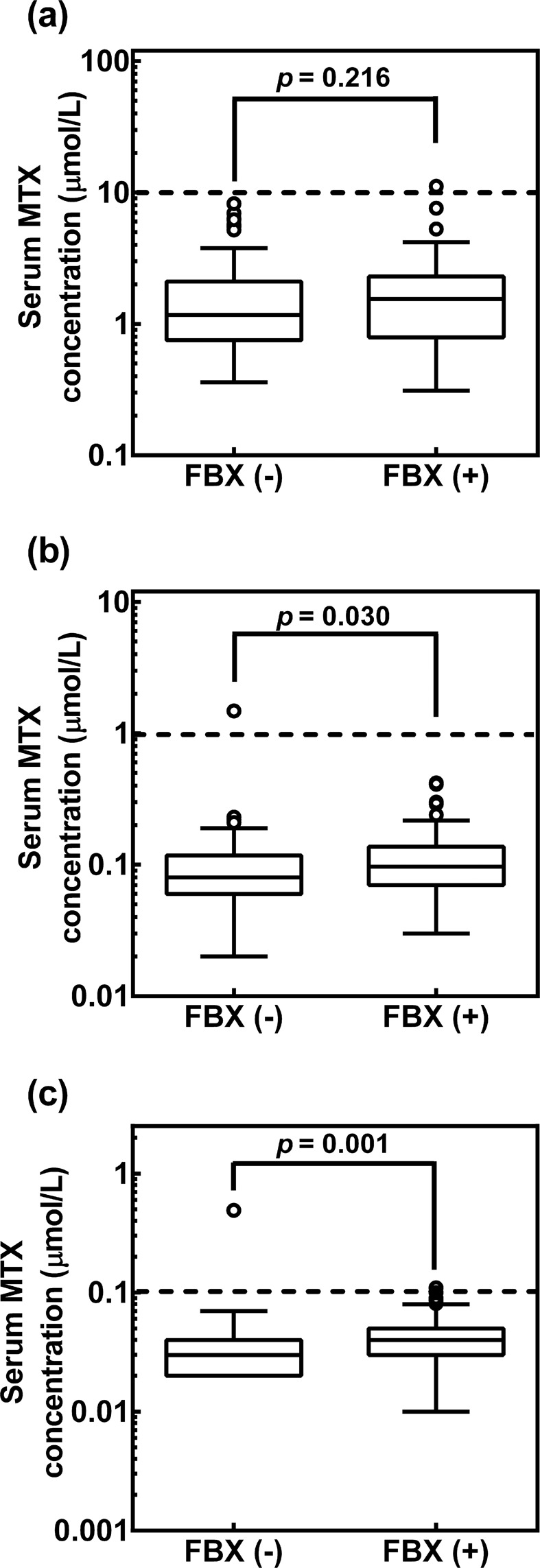
Serum MTX concentrations at 24 h (**a**), 48 h (**b**), and 72 h (**c**) following HDMTX therapy in patients with (72 cycles) and without (72 cycles) receiving FBX. The box and whisker plot represent the median, first and third quartiles, and minimum and maximum values of serum MTX concentration. The open circles represent outliers that are >1.5-times the interquartile range from a quartile. The risk limit values of serum MTX concentration (>10 μmol/L at 24 h, >1 μmol/L at 48 h, and >0.1 μmol/L at 72 h) after MTX administration are indicated by the dotted horizontal lines. Statistical analyses were performed using Mann-Whitney U-test.FBX: febuxostat, MTX: methotrexate.

Figure 2 shows the comparisons of serum MTX concentrations at 24 h (A), 48 h (B), and 72 h (C) following HDMTX therapy between patients receiving FBX (72 cycles) and not receiving FBX (72 cycles). As shown in Fig. 2b,c, the serum MTX levels at 48 and 72 h in patients receiving FBX were significantly higher than in those not receiving FBX (*p* = 0.030 and *p* = 0.001, respectively). On the other hand, there were no significant differences in the serum MTX levels at 24 h between the two groups (*p* = 0.216).

### Analyses on the impact of co-administered drugs on hepatotoxicity following MTX therapy using the FAERS database

After excluding the duplicate reports, 4,133,405 out of 4,413,516 reports in the FAERS database were analyzed. A total of 98,587 cases of MTX-related adverse events were extracted. These were 8,793 cases of MTX-induced hepatotoxicity. The results of the database analyses including reporting ratio of MTX-induced hepatotoxicity, reporting odds ratio (RORs), and 95% confidence interval (CI) in patients receiving FBX, allopurinol, lansoprazole, and rabeprazole are summarized in Table 3. The reporting ratio of hepatotoxicity in patients receiving FBX (29%) was significantly higher than in those not receiving FBX (9%, *p* < 0.001). As for the other drugs observed, the ratio of MTX-indcued hepatotoxicity was not significantly different based on the concomitant use of these drugs. In addition, a positive signal was observed with co-administered FBX (ROR: 4.16, 95% CI: 2.89–5.98) whereas no significant signals were not found with co-administered allopurinol (ROR: 0.95, 95% CI: 0.76–1.19), lansoprazole (ROR: 0.97, 95% CI: 0.82–1.16), and rabeprazole (ROR: 0.88, 95% CI: 0.65–1.20).

**Table 3:** Analyses of the impact of co-administered drugs on hepatotoxicity following MTX therapy using the FAERS database.

Co-administered drug	MTX-induced hepatotoxicity (%)		ROR (95% CI)	*p-*value
	Without drug	With drug		
FBX	8,752/98,445 (9)	41/142 (29)	4.16 (2.89–5.98)	<0.001
Allopurinol	8,708/97,592 (9)	85/995 (9)	0.95 (0.76–1.19)	0.717
Lansoprazole	8,654/96,991 (9)	139/1,596 (9)	0.97 (0.82–1.16)	0.801
Rabeprazole	8,749/98,033 (9)	44/554 (8)	0.88 (0.65–1.20)	0.463

MTX-induced hepatotoxicity presented as cases/(cases + non-cases) (%).

Chi-square test was performed.

CI: confidence interval, FAERS: FDA adverse event reporting system, FBX: febuxostat, MTX: methotrexate, ROR: reporting odds ratio.

### Effect of FBX on cell viability and cytotoxicity after exposure to MTX in HepG2 cells

**Figure 3:**
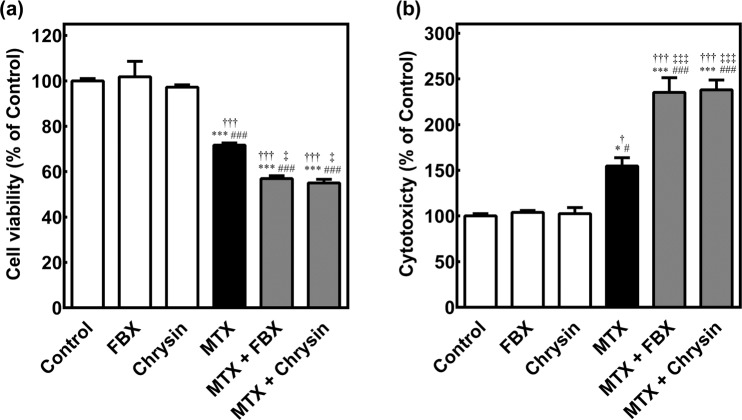
Cell viability (**a**) and cytotoxicity (**b**) after exposure to MTX in HepG2 cells. HepG2 cells were incubated at 37 °C for 24 h with MTX (100 μM) in the presence or absence of chrysin (1 μM) or FBX (0.1 μM). Cell viability and cytotoxicity were determined by MTS assay and cytotoxicity assay, respectively. Cell viability and cytotoxicity in HepG2 cells after treatment of vehicle (control) were set at 100%. Each column represents the mean ± S.E. of three separate experiments. Statistical analyses were performed using Tukey’s multiple comparison test. ^*^p < 0.05, ^***^p < 0.001 compared to control, ^#^*p* < 0.05, ^###^*p* < 0.001 compared to FBX, ^†^*p* < 0.05, ^†††^*p* < 0.001 compared to Chrysin, ^‡^*p* < 0.05, ^‡‡‡^*p* < 0.001 compared to MTX. FBX: febuxostat, MTX: methotrexate.

To assess whether FBX directly enhances hepatotoxicity associated with MTX, the effect of FBX on the cell viability and cytotoxicity in HepG2 cells were examined by MTS assay and cytotoxicity assay, respectively. Figure 3a,b show the cell viability and cytotoxicity in HepG2 cells after exposure to MTX (100 μM) in the presence or absence of chrysin (1 μM) or FBX (0.1 μM) at 37 °C for 24 h. As shown in Fig. 3a, the cell viability was reduced to approximately 72% of that of the control by a single treatment with MTX. Besides, the cell viability was further reduced by co-incubation of FBX or chrysin (57% or 55% of control) with MTX. The cytotoxicity was increased to approximately 154% of that of the control by a single treatment with MTX (Fig. 3b). Co-incubated FBX or chrysin with MTX significantly increased the cytotoxicity (235% or 238% of control). On the other hand, a single treatment with FBX (0.1 μM) or chrysin (1 μM) showed no significant effects on the cell viability and cytotoxicity.

## Discussion

Little is known about the drug interaction between FBX and MTX and the effect of FBX on the development of MTX-related adverse effects in clinical settings. To the best of our knowledge, this is the first study reporting the effect of concomitant FBX on the development of MTX-induced hepatotoxicity through BCRP-mediated drug interaction.

Although Miyata *et al*.^22^ reported that FBX potently inhibits the transport of urate (typical BCRP substrate) in the BCRP-expressing plasma membrane vesicles, the BCRP-mediated drug interaction between FBX and MTX remained to be clarified. As shown in Fig. 1b, inhibition of BCRP-mediated transport of [^3^H]MTX was confirmed by co-incubation of 0.1 and 1 µM FBX. When 40−120 mg of FBX was orally administered in human, the maximum plasma concentration (C_max_) of FBX was reported to be approximately 5.3−13.5 µM^24^. Since the protein binding of FBX is 97.8%^25^, the C_max_ of unbound FBX was estimated to be approximately 0.12−0.30 µM. Although there are no reports on the hepatic concentration of FBX after oral administration of FBX in human, the FBX concentration in the liver is found to be approximately 1.6-fold higher than that in the plasma at 1 h after oral administration of FBX (1 mg/kg) in rats as mentioned in the package insert (Feburic^®^ Tablet, Teijin Pharma Limited). This signifies that the FBX concentration in the liver could be higher than that in the plasma in human. *In vitro* studies demonstrated that co-incubated 0.1 µM FBX with MTX reduced the cell viability and enhanced the cytotoxicity as compared to single MTX exposure in HepG2 cells (Fig. 3a,b). Therefore, these findings reveal that concomitant FBX at a clinical dose should enhance MTX-induced hepatotoxicity by inhibiting BCRP-mediated MTX transport.

To clarify the clinical impact of concomitant FBX on the development of MTX-related adverse effects, we conducted the retrospective chart review in patients received HDMTX therapy and analyzed the FAERS database. Our present study demonstrated that the incidence of hepatotoxicity but neither nephrotoxicity and hematological toxicity in patients with FBX was significantly higher than that in patients without FBX (Table 2). Moreover, analyses using the FAERS database revealed that a positive signal for MTX-induced hepatotoxicity was observed for the concomitant FBX (Table 3). Therefore, these findings suggest that concomitant FBX should enhance the MTX-induced hepatotoxicity in clinical situations.

Since various drug transporters including OATPs, OATs, BCRP, and MRP2 are known to be responsible for the biliary and renal excretion of MTX^8–15^, inhibition of these transporters could be attributable to the higher degree of adverse effects due to increased systemic exposure and/or altered renal and hepatic accumulation of MTX. As shown in Fig. 2, concomitant FBX significantly increased the serum MTX concentration at 48 and 72 h after HDMTX, indicating that concomitant FBX could affect the pharmacokinetics of MTX through inhibition of tissue uptake and/or renal and biliary efflux of MTX via drug transporters. However, little is known about the inhibitory effect of FBX for other transports excluding BCRP. The serum MTX concentrations did not exceed the clinical risk limit values (>1 μmol/L at 48 h and >0.1 μmol/L at 72 h after MTX administration)^18,26^. In addition, the standard prophylactic therapy of HDMTX-related toxicity, such as hydration and calcium folinate rescue was to be administered in all the study patients. Thus, we speculate that concomitant FBX could not increase the incidence of hematological toxicity in spite of increased serum MTX concentrations as shown in Table 2.

Our study shows that FBX increases the incidence of hepatotoxicity in patients receiving HDMTX therapy (Table 2). Because FBX-induced acute liver injury has been reported in some case reports^27,28^, we assessed the causality between FBX and hepatotoxicity in patients receiving HDMTX (Supplementary Table 1). The causality score of FBX administration for the development of hepatotoxicity was “Unlikely” or “Excluded”, indicating that the hepatotoxicity could not be associated with administration of FBX in the present clinical study.

Breedveld *et al*.^14^ investigated the role of BCRP in the clearance of MTX using BCRP knockout mice and mice treated with pantoprazole (a BCRP inhibitor). The absence or inhibition of BCRP did not reduce the urinary excretion but reduced the fecal excretion of MTX after intravenous MTX administration in mice, suggesting that the function and/or activity of BCRP located in the liver predominantly affects the pharmacokinetics of MTX in mice. In human, *ABCG2* mRNA is expressed abundantly in the liver and slightly in the kidney^29^. These findings suggest that concomitant FBX could enhance MTX-induced hepatotoxicity by increasing hepatic accumulation of MTX through inhibition of BCRP in the liver but not in the kidney.

Several case reports and retrospective studies have demonstrated that co-administration of PPIs delayed the elimination of MTX^17,18,30,31^. Following these reports, the statement regarding the precautions for co-administration of PPI with MTX were added in the package insert of MTX in Japan in October 2013. Subsequently, concomitant PPI with MTX was not observed in our clinical study. On the other hand, FBX was approved for medical use in Japan in March 2011, and most patients who received therapy of HDMTX are currently co-administered with FBX for the for prevention of hyperuricemia accompanied by TLS. Therefore, it is assumed the rate of co-administered PPI was significantly higher in patients not receiving FBX than those receiving FBX in Table 1.

In the present study, 17 patients (48 cycles) received PPIs (lansoprazole and rabeprazole) during HDMTX therapy. However, there were no significant differences in the serum MTX levels between patients receiving or not receiving PPI (Supplementary Fig. 1). Besides, no significant difference in the incidence of hepatotoxicity was observed between the two groups (8% vs 10%, *p* = 0.775). In the analyses using the FAERS database (Table 3), co-administration of lansoprazole or rabeprazole with MTX was not found to be a significant risk factor contributing to the development of hepatotoxicity. Thus, these results suggest that concomitant PPI with MTX could not affect the development of MTX-induced hepatotoxicity.

In the present clinical study, TLS was observed in three patients, and none of them received FBX, allopurinol, or PPI. Allopurinol, another xanthine oxidase inhibitor, has no inhibitory effects on the BCRP activity^22^. As shown in Table 3, the co-administration of allopurinol with MTX was not found as a significant risk factor contributing to the development of hepatotoxicity. Although allopurinol is not approved for the prevention of TLS in Japan, it is effective for prevention of hyperuricemia associated with TLS during cancer chemotherapy^32^. Therefore, FBX could be discontinued and/or switched to allopurinol when hepatotoxicity develops with HDMTX therapy.

The present study has some limitations that need to be considered. First, it remains unclear whether the inhibition of FBX on the hepatic BCRP-mediated excretion of MTX mainly contributes to the enhancement of MTX-induced hepatotoxicity because there have been no reports on the inhibitory effects of FBX on the activity of the other transporters. Second, in the analyses using the FAERS database, the effect of FBX on hepatotoxicity following HDMTX therapy was not evaluated because most of the FAERS database had missing data regarding the dosage of MTX used. Finally, it was difficult to exclude the potential effects of other unknown confounders in our retrospective study. Therefore, further studies using *in vitro* and *in vivo* approaches are needed to clarify the detailed mechanism regarding enhancement of MTX-induced hepatotoxicity by FBX. In addition, a prospective study should be also conducted to determine the influence of FBX on the pharmacokinetics of MTX and the development of hepatotoxicity

In conclusion, our study is the first to demonstrate that concomitant FBX enhances hepatotoxicity in patients receiving HDMTX, at least in part, by the inhibition of hepatic BCRP. The present findings provide important information for the safe management of HDMTX therapy in clinical settings.

## Materials and Methods

### Materials

MTX was obtained from Tokyo Chemical Industry Co., Ltd. (Tokyo, Japan). FBX and chrysin were purchased from LKT Laboratories, Inc. (St. Paul, MN) and WAKO Pure Chemical (Osaka, Japan), respectively. [3′,5′,7′-^3^H(N)] MTX ([^3^H]MTX, 29.7 Ci/mmol) was purchased from Moravek Biochemicals Inc. (Brea, CA). All other chemicals used were of the highest purity available.

### Transport study of [^3^H]MTX with human BCRP-expressing plasma membrane vesicles

The human BCRP-expressing plasma membrane vesicles were prepared from baculovirus-infected insect cells, and the control vesicles prepared from the mock-transfected cells were purchased from GenoMembrane Co., Ltd. (Kanagawa, Japan). The transport studies were performed according to the instructions provided by the manufacturer. Briefly, a reaction mixture (final volume, 50 μL) containing 10 μM [^3^H]MTX, 50 mM MOPS-Tris (pH 7.4), 70 mM KCl, and 7.5 mM MgCl_2_, and membrane vesicles (50 μg of protein) was incubated at 37 °C for 5 min in the presence or absence of 4 mM ATP. The transport reaction was terminated by 200 μL of ice-cold wash buffer containing 40 mM MOPS-Tris (pH 7.4) and 70 mM KCl. The stopped reaction mixture was filtered through a UniFilter-96 GF/B (PerkinElmer, Waltham, MA). After washing with ice-cold wash buffer for five times, the filter plates were dried. Then 30 μL scintillation cocktail was added to each well. The radioactivity was counted using a MicroBeta^2^ scintillation counter (PerkinElmer, Waltham, MA). For the inhibition study of chrysin (a potent BCRP inhibitor)^33^ or FBX against BCRP-mediated transport of [^3^H]MTX, the uptake of [^3^H]MTX was measured using BCRP-expressing vesicles with ATP in the presence of 1 μM chrysin or 0.1 and 1 μM FBX. The BCRP-mediated [^3^H]MTX uptake was calculated by subtracting the [^3^H]MTX uptake in the mock-transfected with ATP from that in the BCRP-expressing vesicles with ATP.

### Patients and data collection

A retrospective cohort study was performed with 38 adult hospitalized patients (144 cycles) who received HDMTX therapy for the treatment of diffuse large B-cell lymphoma, intravascular large B-cell lymphoma, and low-grade B-cell lymphoma in the Mie University Hospital between January 2008 and March 2017. The eligible patients received a 2-h intravenous infusion of MTX (2.8–3.5 g/m^2^). For the prevention of adverse effects by HDMTX, all the enrolled patients received hydration with continuous intravenous infusion, urine alkalization with sodium bicarbonate, and intravenous calcium folinate rescue. The patients were excluded if they had missing data and received NSAIDs. The demographic data were extracted from electronic medical records. The serum MTX concentration was determined at 24, 48, and 72 h after the initiation of HDMTX therapy. The co-administered drugs, which may cause potential interactions with MTX, were identified by using Lexicomp^®^ Lexi-Interact^TM^ Online (Lexi-Comp, Inc., Hudson, OH). In addition, we investigated the effects of co-administered of PPIs, granulocyte-colony stimulating factor (G-CSF) agents, and hepatoprotective agents, such as glycyrrhizic acid/amino acetic acid/methionine combined drug, and ursodeoxycholic acid. This study was conducted in accordance with the Declaration of Helsinki. The Ethics Committee of the Mie University Graduate School of Medicine and Faculty of Medicine approved this study (No. H2018–048). Informed consent from participants was obtained through an opt-out method in accordance with the Ethical Guidelines for Medical and Health Research Involving Human Subjects in Japan.

### Evaluation of adverse effects following HDMTX therapy

Hepatotoxicity was defined as alanine aminotransferase (ALT) level ≥5-fold of ULN (upper limit of normal), ALP (alkaline phosphatase) level ≥2-fold of ULN, ALT level ≥3-fold of ULN and direct-bilirubin (D-Bil) level ≥2-fold of ULN in accordance with the criteria for drug-induced live injury^34^. In addition, the clinical pattern, severity, causality assessment, and chronicity of hepatotoxicity were also evaluated. Nephrotoxicity was defined as ≥grade 1 in accordance with the Common Terminology Criteria for Adverse Events version 5.0 (CTCAE) for acute kidney injury. Hematological toxicity was defined as ≥grade 3 of absolute neutrophil count (ANC), platelet (PLT) count, or hemoglobin (Hb) level.

### Analyses on the impact of co-administered drugs on MTX-induced hepatotoxicity using the database of FDA adverse event reporting system

Data consisting of patient demographics and administration information (DEMO), drug/biologic information (DRUG), and adverse events (REAC) from July 2014 to December 2017 were obtained from the database released by the FAERS (http://www.fda.gov/). Duplicate reports were excluded in accordance with the FDA recommendations^35^. Data analyses were performed using ACCESS^®^ 2016 (Microsoft, Redmond, WA). The data associated with MTX administration were extracted. Disease names were defined using the Medical Dictionary for Regulatory Activities (MedDRA/J) ver 21.1., and the standardized MedDRA Query (SMQ) for drug-related hepatic disorders-comprehensive search (SMQ code:20000006) was used. The impact of co-administered drugs on the development of MTX-induced hepatotoxicity was evaluated using the RORs. To calculate the ROR, hepatotoxicity associated with MTX and all other reported adverse events associated with MTX were defined as “cases” and “non-cases”, respectively. The RORs were calculated from two-by-two contingency tables of counts with or without FBX, allopurinol, lansoprazole, and rabeprazole. RORs were expressed as point estimates with 95% confidence interval (CI). The positive signals were defined as the lower limit of 95% CI for the ROR of >1^36^.

### Cell culture

Human hepatocellular carcinoma cell line HepG2 cell was obtained from RIKEN BioResource Research Center (Tokyo, Japan). HepG2 cells were cultured in Dulbecco’s modified Eagle’s medium supplemented with 10% fetal bovine serum and were used between passage numbers 26 and 33. These cells were maintained at 37 °C in presence of 5% CO_2_ in a humidified atmosphere.

### Determination of cell variability and cytotoxicity in HepG2 cells

HepG2 cells have low levels of cytochrome P450 enzymes compared with primary hepatocyte^37^ whereas the expression levels of BCRP in HepG2 cells is comparable to that in primary hepatocyte^38^. Therefore, the inhibitory effects of BCRP-mediated efflux of MTX against cell variability and cytotoxicity was evaluated in HepG2 cells according to a previous report^39^. Briefly, HepG2 cells were seeded on a 96 well-plate at a density of 2.5 × 10^4^ cells/well. After 24 h of seeding, HepG2 cells were incubated with the culture medium containing MTX in the presence or absence of 1 μM chrysin (a potent BCRP inhibitor)^33^ or 0.1 μM FBX. After 24 h of exposure, the cell viability in HepG2 cells was determined using CellTiter 96^®^ AQueous One Solution Cell Proliferation Assay (Promega, Madison, WI) following the instructions provided by the manufacturer. The absorbance was measured at 490 and 620 nm with the iMark^TM^ Microplate Absorbance Reader (Bio-Rad, Hercules, CA). The cytotoxicity in HepG2 cells was determined using CytoTox-Glo™ Cytotoxicity Assay (Promega, Madison, WI) following the instructions provided by the manufacturer. The luminescence was measured with 2300 Multiplate Reader ARVO^TM^ X2 (PerkinElmer, Waltham, MA). The cell viability and cytotoxicity in HepG2 cells after treatment of vehicle (control) were set at 100%.

### Statistical analyses

The results of the *in vitro* experimental data are expressed as the mean ± S.E. Statistical comparisons for multiple groups were performed using one-way analysis of variance followed by Dunnett’s test or Tukey’s multiple comparison test for the results of the *in vitro* study. For the clinical study, statistical comparisons between the two groups were performed using the Mann-Whitney U-test and Fisher’s exact test (or Chi-square test) for continuous and categorical variables, respectively. All statistical analyses were performed using GraphPad Prism 6.07 (GraphPad Software Inc., San Diego, CA). The significance was established at a *p*-value < 0.05.

## Supplementary information

Supplementary Figure 1.

Supplementary Table 1.
