# Supplementary material for: Concomitant febuxostat enhances methotrexate-induced hepatotoxicity by inhibiting breast cancer resistance protein
Source: Sci Rep. 2019 Dec 30;9:20359. doi: 10.1038/s41598-019-56900-2 (PMC6937279; doi:10.1038/s41598-019-56900-2)
Supplement: Supplementary file 1 — Supplementary Figure 1. [file 41598_2019_56900_MOESM1_ESM.pdf]

Concomitant febuxostat enhances methotrexate-induced hepatotoxicity by inhibiting breast cancer resistance protein

Kenji Ikemura[1*#], Shun-ichi Hiramatsu[2#], Yuri Shinogi[1], Yusuke Nakatani[1], Isao Tawara[3], Takuya Iwamoto[1,2], Naoyuki Katayama[3], and Masahiro Okuda[4]

[1]Department of Pharmacy, Mie University Hospital, Tsu, Mie 514-8507, Japan

[2]Department of Clinical Pharmacy and Biopharmaceutics, Mie University Graduate School of Medicine, Tsu, Mie 514-8507, Japan

[3]Department of Hematology and Oncology, Mie University Graduate School of Medicine, Tsu, Mie 514-8507, Japan

[4]Department of Pharmacy, Osaka University Hospital, Suita, Osaka 565-0871, Japan

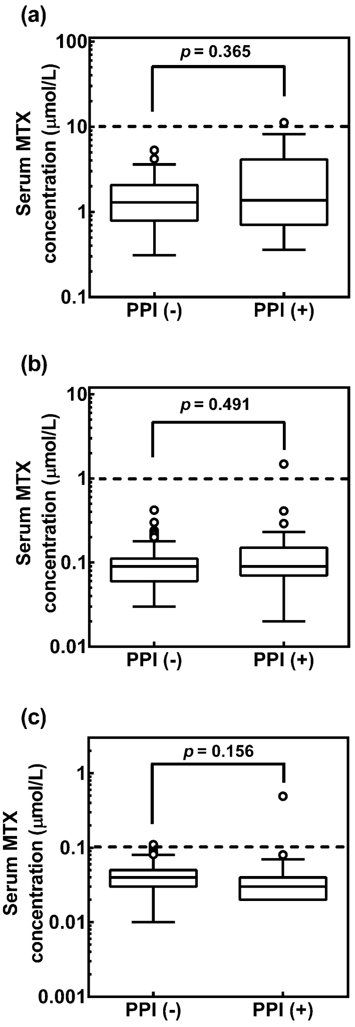

Supplementary Figure 1. Serum MTX concentrations at 24 h (a), 48 h (b), and 72 h (c) following HDMTX therapy in patients with (48 cycles) and without (96 cycles) receiving PPI. The box and whisker plot represent the median, first and third quartiles, and minimum and maximum values of serum MTX concentration. The open circles represent the outliers that are > 1.5-times the interquartile range from a quartile. The risk limit values of serum MTX concentration (> 10 μmol/L at 24 h, > 1 μmol/L at 48 h, and > 0.1 μmol/L at 72 h) after MTX administration are indicated by the dotted horizontal lines. Statistical analyses were performed using Mann-Whitney U-test. MTX: methotrexate, PPI: proton pump inhibitor
