# Supplementary material for: Concomitant febuxostat enhances methotrexate-induced hepatotoxicity by inhibiting breast cancer resistance protein
Source: Sci Rep. 2019 Dec 30;9:20359. doi: 10.1038/s41598-019-56900-2 (PMC6937279; doi:10.1038/s41598-019-56900-2)
Supplement: Supplementary file 2 — Supplementary Table 1. [file 41598_2019_56900_MOESM2_ESM.pdf]

Concomitant febuxostat enhances methotrexate-induced hepatotoxicity by inhibiting breast cancer resistance protein

Kenji Ikemura[1*#], Shun-ichi Hiramatsu[2#], Yuri Shinogi[1], Yusuke Nakatani[1], Isao Tawara[3], Takuya Iwamoto[1,2], Naoyuki Katayama[3], and Masahiro Okuda[4]

[1]Department of Pharmacy, Mie University Hospital, Tsu, Mie 514-8507, Japan

[2]Department of Clinical Pharmacy and Biopharmaceutics, Mie University Graduate School of Medicine, Tsu, Mie 514-8507, Japan

[3]Department of Hematology and Oncology, Mie University Graduate School of Medicine, Tsu, Mie 514-8507, Japan

[4]Department of Pharmacy, Osaka University Hospital, Suita, Osaka 565-0871, Japan

Supplementary Table 1. **Characteristics of hepatotoxicity in cases defined as acute drug-induced liver injury**

Case No.	Concomitant FBX	Clinical pattern	Severity	RUCAM-based causality		Chronicity	AIH
				MTX	FBX		
1	–	Hepatocellular	Mild	Probable	–	–	–
2	–	Hepatocellular	Mild	Probable	–	–	–
3	+	Hepatocellular	Mild	Probable	Unlikely	–	–
4	+	Hepatocellular	Mild	Highly probable	Unlikely	–	–
5	+	Hepatocellular	Moderate	Probable	Unlikely	–	–
6	+	Hepatocellular	Moderate	Probable	Unlikely	–	–
7	+	Hepatocellular	Mild	Probable	Unlikely	–	–
8	+	Hepatocellular	Mild	Probable	Unlikely	–	–
9	+	Hepatocellular	Mild	Probable	Excluded	–	–
10	+	Hepatocellular	Mild	Probable	Unlikely	–	–
11	+	Hepatocellular	Mild	Probable	Unlikely	–	–
12	+	Hepatocellular	Mild	Probable	Unlikely	–	–
13	+	Hepatocellular	Mild	Probable	Unlikely	–	–
14	+	Hepatocellular	Mild	Probable	Unlikely	–	–

Clinical pattern, severity, causality assessment, chronicity of hepatotoxicity, and drug-induced autoimmune hepatitis were assessed using the Council for International Organizations of Medical Science scale.

AIH: autoimmune hepatitis, FBX: febuxostat, MTX: methotrexate, RUCAM: Roussel Uclaf Causality Assessment Method.
